# A critical review of the reporting of reflexive thematic analysis in *Health Promotion International*

**DOI:** 10.1093/heapro/daae049

**Published:** 2024-05-28

**Authors:** Virginia Braun, Victoria Clarke

**Affiliations:** School of Psychology, The University of Auckland, Private Bag 92019, Auckland Mail Centre 1142, New Zealand; School of Social Sciences, University of the West of England, Coldharbour Lane, Bristol BS16 1QY, UK

**Keywords:** Big Q qualitative, coding, methodological congruence, saturation, small q qualitative, theme, topic summary

## Abstract

Using the concept of methodological congruence—where the different elements of a study ‘fit’ together—we explore both problematic and good practice in (reflexive) thematic analysis (TA) as reported in *Health Promotion International (HPI)*. Aligning with the importance we place on ‘owning your perspectives’ we situate this exploration in relation to our understanding of the variation in approaches to TA and qualitative research more broadly. This contextualization is necessary for highlighting why we judge practices to be in/congruent, and to facilitate more knowing congruence in future research. We critically reviewed a ‘sample’ of 31 papers published in *HPI* between 2010 and 2023 citing Braun and Clarke as reference for TA. We overview a range of problematic and good features of the use of TA in *HPI*, before focusing on two domains that seemed to present key challenges: theory and themes. Methodological incongruence can occur when postpositivist values and practices unwittingly creep into ostensibly non-positivist TA; we encourage thoughtfully and what we term ‘knowing’ consideration of theory, and quality practices and criteria. Methodological incongruence can also occur through mismatched conceptualizations of themes—notably, the use of ‘topic summaries’ as themes for reflexive TA (and fragmented thematic structures with ‘thin’ themes). We provide examples from the reviewed papers to demonstrate good practice in researcher reflexivity, articulation of theoretical and methodological frameworks and congruent themes. However, mindful of power dynamics, we only discuss problematic practice in general terms, to protect author anonymity. To facilitate thoughtful, quality TA—of all kinds—we provide eight pointers for researchers (and reviewers) to guide quality practice, and facilitate the use of concepts, procedures and criteria that promote knowing methodological congruence.

**Contribution to Health Promotion**

- This article aims to support researchers in improving the quality and coherence of their thematic analysis (TA) research by identifying both common problems and good practices.
- The article critically reviews 31 papers using TA published in *Health Promotion International* that cite Braun and Clarke.
- The review also aims to support manuscript reviewers in identifying good and problematic practice and providing researchers with constructive and coherent feedback on their TA research.
- We provide recommendations for good practice—including choosing an approach to TA most appropriate to the purpose and values of the research.

## A CRITICAL REVIEW OF THE USE OF REFLEXIVE THEMATIC ANALYSIS IN *HEALTH PROMOTION INTERNATIONAL*

Thematic analysis (TA), including the reflexive TA approach we have outlined (e.g. Braun and Clarke, 2006, 2022), is widely used in qualitative health promotion research, including that published in *Health Promotion International (HPI)*. We first wrote about TA for a psychology audience, but reflexive TA has become widely taken up beyond psychology. (In March 2024, our initial 2006 paper had over 190,000 Google Scholar citations.) Our aim was to develop an approach to TA which aligned with our *fully* qualitative or non-positivist orientation to qualitative research. Not all approaches to TA (developed prior or since) can be so characterized (Finlay, 2021). As such, TA is best thought of as a *family* of methods, with shared characteristics, alongside important differences in underlying philosophy, concepts and procedures. These divergences matter for research practice and for quality, but—evidenced by our reviews of published TA research (Braun and Clarke, 2021c), including in health (Braun and Clarke, 2023a, 2023b, 2024)—it appears the methodological consequences of them are not well understood, and what we have referred to elsewhere as ‘unknowing’ practice (Braun and Clarke, 2023b) is common. Unknowing practice is the flip side of what we consider to be good—*knowing*—practice: a researcher who is deliberative, thoughtful and reflexive in their choices and articulation of their research. They don’t uncritically accept directives for ‘good practice’ (including ours in this article!), but, drawing on methodological literature, reason through whether particular concepts and practices are congruent with their research. A knowing researcher then is also oriented to methodological congruence—the conceptual alignment or ‘fit’ between different elements of a research project—as we discuss further below.

In this *Perspectives* article, we briefly critically review a selection of papers published in *HPI*, in order to support: (i) quality practices for health promotion researchers using TA; and (ii) quality, constructive and congruent reviewing of manuscripts reporting TA. We first briefly demarcate the domains of qualitative research and TA, to contextualize and ground the problematic (and good) practices we identified through our review. Our review then homes in on two particular problematic aspects: the gnarly problem of theory and the lack of clarity and congruence in theme conceptualization. We finish with some key recommendations for quality practice.

## QUALITATIVE RESEARCH AND THEMATIC ANALYSIS: DEFINING AND MAPPING THE TERRAIN

The term *fully* qualitative (which we used above) evokes a distinction between understanding qualitative research in (i) proceduralist or (ii) values-based ways. A proceduralist definition equates it with the use of qualitative *techniques* for data generation and analysis; a values-based definition crucially refers to a wider distinctly qualitative values framework, *within* which qualitative approaches to data generation and analysis are used. The idea of research values offers a simplified catch-all for the philosophical meta-theoretical assumptions that ground and give validity to research: paradigms (the broad world views, norms and assumptions underpinning research); ontologies (theories of being and reality); epistemologies (theories of knowledge and knowledge production) and so on. To us, qualitative research has two key components: (i) research techniques, procedures and practices and (ii) the values that underpin those practices, and determine things like what constitutes meaningful knowledge and knowledge production, what data give researchers access to (e.g. contextually situated lived experience; discursive practices), and what constitutes good research practice.

### Big Q and small q qualitative

An example of a proceduralist definition of qualitative research is the use of ‘non-quantitative methods to contribute new knowledge and provide new perspectives on healthcare’ (Tong *et al*., 2007, p. 350), provided by the influential Consolidated Criteria for Reporting Qualitative Research (COREQ) checklist. Another term for this version of qualitative research is *small q* qualitative (Kidder and Fine, 1987), where, in the absence of an explicitly articulated values framework, research tends to default to disciplinary dominant norms—typically some variant of positivism and/or realism. Kidder and Fine’s term for values-based qualitative research is *Big Q* qualitative. Although whether there is one *unifying* set of underlying Big Q research values is debated, Big Q is often understood as non- (or anti-)positivist, theorizing knowledge production as partial, situated and contextual, and acknowledging, and valuing, researcher subjectivity as a resource for research rather than a problem to be managed. These contrast with the post-positivist aspiration for objectivity, ensuring the accuracy and reliability of analysis and keeping researcher ‘bias’—understood as researcher subjectivity threatening and distorting objectivity—in check.

### Experiential and critical qualitative

In mapping the terrain of *Big Q* qualitative, we find it useful to distinguish between ‘experiential’ and ‘critical’ orientations (Braun and Clarke, 2013, 2022)—umbrella terms that designate different orientations for and ‘tasks’ of research. Experiential—probably the vast majority of qualitative research, especially in health—designates qualitative approaches that take a broadly empathic interpretative orientation in exploring, understanding and interpreting human experience, which is typically viewed as socially embedded and/or contextually located. Language becomes a tool for accessing such experience. Critical approaches in contrast interrogate the social construction of meaning and treat language as shaping reality not just conveying reality. In critical qualitative research, the focus moves away from (socially embedded) individual experience and sense-making to consider sense-making as a social practice. This research could be described as theoretically/conceptually critical, as the term ‘critical qualitative’ is (confusingly) also used to describe research with an overt social justice agenda and an interest in power and the social world—this latter version of critical qualitative is particularly common in the USA in our discipline of psychology (Levitt *et al*., 2021). That quite different traditions use the same term highlights the importance of researchers carefully theoretically locating their approach to qualitative research in a particular study, with descriptions grounded in the relevant literature.

### Diversity within thematic analysis—coding reliability, reflexive and codebook approaches

Approaches to TA range from small q to Big Q—our approach offers the latter (although we did not explain it in those terms initially). As we have witnessed our approach used in ways that do not align with its underpinning values, we have taken care to more clearly differentiate the approach, and its values and practices—hence the (newish) name *reflexive* TA, emphasizing the inherent and inescapable subjectivity of the method (which is a good thing!), and the researcher’s active role in knowledge generation (Braun and Clarke, 2019). We have also sought to clearly (from our perspective) map the terrain of TA, locating our and other approaches in this landscape (see Braun and Clarke, 2022)—we hope constructionist readers forgive us for the realist metaphor. We now differentiate TA into three broad clusters (initially it was two; Finlay [2021] similarly makes a bipartite differentiation):

- TA methods that offer a small q ‘coding reliability’ approach (e.g. Boyatzis, 1998; Guest *et al.*, 2012). With a foothold in positivism, coding reliability TA typically offers structured coding and procedures for theme *identification* oriented to ensuring and demonstrating the accuracy and reliability of coding.
- Big Q ‘reflexive TA’ approaches, like ours. These non-positivist approaches are characterized by organic and open procedures for coding and theme *development* that centre the researcher’s interpretative engagement with the data. The language of identification versus development of themes conveys a fundamentally different way of imagining themes: either things that can be ‘found’, that exist and that we seek to know, or things that are produced, an *outcome* of the analysis process, unknowable *before* the analytic work is done.
- (3) Our final cluster of TA methods is those we term ‘codebook TA’. These tend to have a relatively structured coding approach (like small q/coding reliability TA) but, being founded more in qualitative values (like Big Q/reflexive TA), they do not tend to treat ‘coding accuracy’ as key for quality assurance. The label ‘codebook TA’ is a useful differentiating tool—but it is important to note that such approaches typically have non-TA names, such as framework analysis—developed for applied policy research (Ritchie and Spencer, 1994), and increasingly popular in health research (see Smith and Firth, 2011)—and template analysis (King and Brookes, 2016)—developed in psychology, and influential in organizational research.

## KNOWINGNESS AND METHODOLOGICAL CONGRUENCE FOR QUALITY THEMATIC ANALYSIS

In reviewing published research, our intent is not to determine if ‘the rules’ have been followed, which would reflect a rather technical orientation to qualitative research, with narrow and fixed ideas of perfection (see Chamberlain, 2000). Instead, we seek to understand methods-as-used, to understand incongruency, and where ‘gaps’ in understanding appear to be, so that we can better advise researchers for quality scholarly practice. For us, quality in TA is not determined by *adherence* to any particular version, or following ‘the recipe’ for TA, so much as practising and reporting in a way that is knowing and methodologically congruent—or *knowingly* incongruent (we have previously used the terms methodologically coherent and incoherent in our methodological scholarship but now prefer the less negative connotations of incongruent). As previously noted, a knowing researcher is one who strives to be informed about the conceptual foundations of particular methods and research practices, and the logics of their inquiry. It is someone who makes deliberative choices (starting from being aware there are choices *to make*, and recognizing how these matter) and is reflexive about their role in knowledge production. *Knowingness* captures understanding of what you are doing, why you are doing it, and *how* you are doing it makes (conceptual) sense.

### Methodological congruence as a key quality marker

Knowing practice is crucial for methodological congruence. Research is methodologically congruent when different components ‘fit’ together—such components include: research question(s); ontology; epistemology; methodology; explanatory theory; methods; quality standards and practices; conceptualization of language and researcher subjectivity; assumptions about what data access and so on. Congruence can be imagined via an analogy of hosting an evening meal (dinner) for friends or family—within whatever cultural context, there will be foods designated as ‘appropriate’ and ‘inappropriate’ for dinner, and a congruent meal would include and exclude on that basis. In a UK context, for instance, porridge or muesli would be incongruent if they appeared on the dinner table, whereas pasta and salad would be congruous. *Knowing* practice involves presenting your guests with either appropriate foods, or offering an explanation of why an inappropriate food is there (which they may judge to be justified, or not—a fancy porridge, such as UK celebrity chef Heston Blumenthal’s infamous snail porridge, may ‘work’ in some contexts).

Across TA approaches, different research practices are congruent or incongruent, depending on the particulars of the approach. Knowingness involves understanding this, and considering in/congruence around the practices used. Take data saturation, often presented as the ‘gold standard’ for determining ‘sample size’ in qualitative research, and included in reporting checklists like COREQ (Tong *et al*., 2007). Knowingness would involve a researcher reasoning through the theoretical assumptions embedded in the concept of data saturation to determine whether it is a good ‘fit’ for their research (e.g. Varpio *et al*., 2017; Braun and Clarke, 2021c). Notions of congruence (and Levitt *et al.*, 2018 analogous concept of methodological integrity) often do not feature in quality and reporting checklists and standards, which is one of the reasons we often find their usefulness is limited, especially in relation to Big Q/reflexive TA.

## REVIEWING THEMATIC ANALYSIS PAPERS IN *HEALTH PROMOTION INTERNATIONAL*

We used the journal search function (on the default relevance setting) to search for ‘thematic analysis’. We selected the first 31 papers citing any of our TA writing; papers did not have to report a reflexive TA or claim to ‘follow’ the six-phase analytic process we have outlined. (We intended to review 30 papers—a nice round number—but miscounted and ended up with 31. So we leant into the stereotype that qualitative researchers do qualitative research because they can’t do numbers, and kept the 31!) This is because it can be difficult to determine in published papers what, if any, approach has been used, because of a lack of or limited description of the researcher’s analytic process (Braun and Clarke, 2023a). The papers were published between 2010 and 2023, with most (*n* = 27) published in the 5 years from 2019 to 2023. Being mindful of power dynamics, we follow Smith’s (2011) use of anonymity, describing in *general* terms the hallmarks of problematic practice. However, as there *is* value in real-world examples, we provide some examples of good practice from the reviewed papers (it’s important to note, however, that few papers were consistently ‘good’; papers often contained both good *and* problematic practice). Before focusing specifically on two domains (methodological incongruence and apparent theoretical unknowingness; theme (mis)conceptualization), we briefly summarize and evaluate the use of TA in *HPI*.

## CHARACTERISTICS AND EVALUATION OF THEMATIC ANALYSIS RESEARCH IN *HEALTH PROMOTION INTERNATIONAL*

### Research design and research questions

Most papers reported standalone qualitative studies—some were part of wider studies including some mixed methods designs—and across the papers, a variety of research questions types were addressed. Referencing a typology of ‘qualitative research questions’ we have developed (Braun and Clarke, 2013), TA was used to address questions centred on lived experiences, participants’ perceptions of and perspectives on particular phenomena, participants’ practices or behaviours in relation to particular phenomena, influencing factors (‘barriers and facilitators’—to the implementation of a particular policy, intervention or health behaviour—was a very common focus), and the representation of particular phenomena in particular contexts. Health promotion researchers are clearly harnessing the potential that TA offers for addressing a wide range of research questions (Braun and Clarke, 2022). The flexibility TA offers was also evident with regard to data generation methods and data sources used, including interviews of various modalities, focus groups, secondary sources, qualitative surveys, observation, journal writing, photomethodology and stakeholder reports. Multimethod designs were common, with more studies than not having multiple (qualitative or qualitative and quantitative) data sources.

### Thematic analysis approach used

Our evaluation of the TA research published in *HPI* was not radically different from that we have reviewed in other journals (e.g. Braun and Clarke 2023a, 2023b, 2024). All of the papers reported some kind of TA or the results of a TA adjacent method; 29 described the method used as TA and two papers (published in 2022) as reflexive TA. The most commonly cited source was our original paper (Braun and Clarke, 2006)—often seemingly treated as a generic reference for any TA. In 22 papers, this was the sole (Braun and Clarke) source cited. In three other papers, including the two describing using *reflexive* TA, more recent (Braun and Clarke) sources were cited (e.g. Braun and Clarke, 2013, 2017, 2021a, 2021b, 2021c, 2022; Clarke and Braun, 2017; also Byrne, 2021). (The way we think about and articulate our approach has evolved, and will likely continue to do so, so reading more recent work is important if using reflexive TA.) Some papers (also) cited other TA approaches (e.g. Fereday and Muir-Cochrane, 2006; DeCuir-Gunby *et al*., 2010; Patton, 2015) or other methodologies (e.g. grounded theory; Strauss and Corbin, 1990; Charmaz, 2006). (Although some still advocate for ‘thematic coding’, where grounded theory coding procedures and other analytic techniques are used to develop themes from qualitative data (e.g. Flick, 2018), as there is now a plethora of methodological literature for TA *approaches*, we think it is important to justify the use of techniques from another methodology over the use of methods specifically developed *for* TA.) In a few examples, authors appeared to have developed and used an idiosyncratic TA approach by drawing on guidance on conducting TA or another approach written by several different authors. Typically, any philosophical and procedural differences across sources from different authors/approaches were not acknowledged or discussed (and a rationale for a combined or ‘new’ approach was not provided). Indeed, there was considerable variation and ‘messiness’ in both the described process of doing, and language of reporting, analysis—such as ostensibly claiming to ‘follow’ our approach but then describing something different; or reporting the use of our method, but using the language and concepts of grounded theory, such as line by line coding (see Braun and Clarke, 2021a). The variation in practice ascribed to Braun and Clarke (2006) was so great that sometimes it seemed likely that some authors had not actually read the paper (see also Braun and Clarke, 2023a).

### The many questions of thematic analysis—semantic and latent coding, inductive and deductive analytic orientations

There is a need to think about and make decisions in *using* TA as an analytic method—we have described these as the ‘many questions of TA’ (see Braun and Clarke, 2006). Such decisions relate to different possible orientations to and treatments of data, coding and analysis. Only a minority of authors described a coding orientation (reporting they coded data semantically or used a mix of semantic and latent coding). Those who noted an analytic orientation most frequently described an inductive approach; some described a *combination* of inductive and deductive. A deductive orientation was equated with practices aligned more with coding reliability and codebook TA approaches (e.g. Boyatzis, 1998) than reflexive TA, such as: using existing theoretical constructs as themes, and organizing the data into these themes; developing a coding framework using an existing theory/theoretical model; developing codes from the research questions/aims of the study or using themes from existing literature to guide coding. In reflexive TA, a deductive coding orientation means that theory offers an interpretative *lens* for reading and making sense of data, so it shapes the focus of analysis rather than evoking (implicitly) the hypotheticodeductive model. For example, in a paper exploring policymakers’ perspectives on school-based health initiatives in Victoria, Australia, Meiklejohn *et al*. (2020, p. 1463) described how they used interpretive policy analysis as a lens for analysing their data. This approach ‘explore[s] the multiple meanings or perspectives of policies and how these meanings are communicated through the policies’. Defining the coding and inductive/deductive orientations we use is important both for ‘reflexive openness’ (Jacobs *et al*., 2021) and for centring considerations of methodological congruence.

### Methodological frameworks

In the reviewed papers, generally, there was little discussion of a broader methodological framework, although some described using TA within a grounded theory methodology, and some noted the use of a ‘descriptive’ or ‘generic’ qualitative approach. Some referenced TA *as* methodology—which we highlight, because while language is slippery, to us, TA is more method than methodology. We distinguish methods as not predetermining or delimiting things like theoretical frameworks (onto/epistemology), or offering guidance (or directives) around ideal and appropriate research questions, dataset/participant group constitution and size, data generation methods and so on—methods are not an ‘off-the-shelf’ package for a whole research project. Some analytic approaches—like grounded theory, interpretative phenomenological analysis and discourse analysis—are more methodological, with research elements from theory to research question delimited or determined (Chamberlain, 2012; Braun and Clarke, 2021a). However, TA is not *pure* method, as different TA approaches are anchored by broad and different (qualitative) research values: we like how Finlay’s (2021) distinction between scientifically descriptive and artfully interpretative TA conveys this. With TA not being a methodology but a method(ish) approach, the underpinning ontological and epistemological assumptions, guiding theoretical frameworks and other specific elements like the approach to ‘sampling’ and data generation, need to be specifically *selected* for the project. To continue the dinner for friends or family analogy we used earlier, ‘off-the-shelf’ methodologies can be thought of as akin to serving a premade lasagne, whereas a method(ish) approach like TA is more like serving a lasagne you’ve made yourself. In both instances, the basic components of the dish (pasta, white sauce, tomato sauce, cheese) are the same, but when you make your own lasagne, you select the different components (e.g. gluten-free or wheat pasta, dried or fresh, a meat- or vegetable-based tomato sauce with a glass of red wine added or not, cow’s milk or vegan cheese). And ideally, you select and combine them in a way that makes for a harmonious and tasty dish!

### Researcher reflexivity and knowingness

Finally, we come to evident ‘knowingness’ in the reviewed papers. In general, there was relatively little evidence of researcher reflexivity, and/or researchers striving to ‘own their perspectives’ (Elliot *et al*., 1999) and positioning in the reviewed papers. (Publishing norms and styles can actively work against the inclusion of such material in published work, although it can be conveyed more subtly through explicit discussion of, for instance, (meta)theory.) Freeman *et al*. (2017, p. 1051) offered a nice example of good practice in researcher reflexivity. In research exploring UK university students’ perceptions of walking through and being with nature, the first author included reflection on their own walking and solo experience (WSE):

Within the current research I acknowledge that my beliefs, experiences and interests influenced my decision-making and actions and thus shaped the research. My experiences of journeying in ‘wild’ landscapes, on my own and with groups, led me to conduct this research and influenced my ideas about peoples’ behaviour in nature. These experiences have provided me with the knowledge, skill and confidence to create and lead my own WSE which inevitably changes the dynamics of the research process and relationship that can be formed with participants, e.g. sometimes my leadership role and previous outdoor experiences made it easier to build rapport and at other times it did not. I acknowledge that research findings are my own experiential account of the research, which is contingent, provisional, partial, restricted and local (Miller *et al.*, 2008); I included wholeheartedly my own subjectivity in the research.

Harrison *et al*. (2020, p. 1324) provided one of the few other examples of explicit researcher reflexivity. In a media framing analysis of the representation of climate change and health in two New Zealand media sites, they briefly acknowledged how their analysis was shaped by assumptions about the topic and the coverage on one site:

Engaging in a process of reflexivity, we acknowledge that our analysis is informed by our understanding of what defines ‘health’, our belief in the importance of the natural environment for health and well-being, and a pre-held hypothesis that the commercially owned [*New Zealand Herald Online*] would lean towards sensationalized, de-contextualized framing.

Reflexive openness most broadly in TA includes the researcher ‘owning’ their theoretical underpinnings and orientation, which we consider now.

## REFLEXIVE NON-OPENNESS AND THE SEEMINGLY GNARLY PROBLEM OF THEORY

There is no such thing as atheoretical qualitative research—all research is anchored in assumptions and theorizations about the nature of reality, what constitutes meaningful knowledge and what data provide access to. These broad or meta-theoretical considerations need to be concurrent to any (additional) discussion of explanatory theory or framing theoretical models that inform analysis—which included, in the reviewed papers, interpretative policy analysis, media framing, social cognition, psychosocial and ecological models.

### Experiential and critical qualitative

Considering the question of data, we categorized most papers as broadly experiential—evident through references to a ‘subjectivist epistemology’ or an interpretivist paradigm when philosophical meta-theory was mentioned—which conceives data as a way into participant worlds and experiences. A critical orientation (both social construction of meaning and social justice versions) was evident in some papers. *One* referenced constructionism and theorized language as performative and action-oriented—with data understood as giving access to meaning-*making* not interior realities. Some articles noted constructivism, constructionism and relativism as ontologies/epistemologies—which starts to make theory more explicit. But reviewing these as critical scholars, the descriptions and/or enactment of claimed theory in the research was often closer to contextualism/critical realism. And we noted the conflation of constructionism and constructivism, reflecting existing messiness (geographic and disciplinary variation) and interchangeable use of these terms/frameworks (by some). This is an area where more explicit articulation of the consequences of theory for research is important (drilling down into the literature suggests important differences between constructivism and constructionism, with the theorization of language as active and performative a distinct characteristic of constructionism; see Braun and Clarke, 2022).

### The seemingly unknowing dominance of positivism and realism

Although most papers included little or no deliberative discussion of philosophical meta-theory, theory and its influence were present implicitly, and often in ways which worked to produce reflexive non-openness and methodological incongruence. The persistently strong tentacles of (versions of) positivism and realism on the (qualitative) social and health sciences were evident through the deployment of concepts and practices widely advocated as, and perhaps (therefore) assumed to be, theoretically neutral/trans-theoretical. Reported practices or research concerns included: saturation; (researcher) bias; theme consensus/agreement; member checking; concern for the accuracy and reliability of data, coding or interpretation; intercoder reliability; (lack of) representativeness, lack of (statistical) generalisability; triangulation and more. The theoretical foundations (and therefore delimited applicability) of these were typically only expressed by authors orienting to or explaining *why* they hadn’t used concepts and practices like saturation or theme agreement (for critical discussions of many of these, see Braun and Clarke, 2013, 2021c, 2022; Smith, 2017; Varpio *et al*., 2017, 2021; Smith and McGannon, 2018). For example, both Buckler *et al*. (2023) and McGrath *et al*. (2022) described methodologically congruent choices/practices and their rejection of methodologically incongruent practices (which were commonly used in other papers reviewed). Buckler *et al*. described the involvement of the co-authors as ‘critical friends’ to encourage reflexivity and quality practice:

Throughout data collection, analysis and writing of the manuscript, co-authors also acted as ‘critical reviewers’ (Smith and Sparkes, 2016) to encourage deep exploration and alternative interpretations of the data and as a step recommended to overcome recognized limitations of member checking, inter-rater reliability and universal criteria for enhancing trustworthiness (Smith and McGannon, 2018). (p. 3)

McGrath *et al*. explained their use of the concept of information power to determine the number of participants:

in keeping with the principles and ethos of reflexive thematic analysis, the concept of ‘data saturation’ was viewed as inconsistent with the values and assumptions of reflexive thematic analysis and more consistent with a straightforward realist ontology (Braun and Clarke, 2021c). Rather, assumptions of reflexive thematic analysis align with the view that when research is situated as a reflexive practice of knowledge generation, there is always potential for new insights or understanding (Braun and Clarke, 2021c). For this reason, the concept of information power was applied—where the larger information power the sample holds the less participants are needed (Malterud *et al.*, 2016). (p. 4)

They also explained why they had not sought to establish the reliability of their coding:

This approach to coding is organic rather than reliant on any particular coding framework, with the generation of themes being the final outcome of data coding and iterative theme development (Braun and Clarke, 2021a). For this reason demonstrating coding reliability is illogical as researcher subjectivity is conceptualized as a resource for knowledge production (Braun and Clarke, 2021b). Rather, rigor in terms of analysis was sought through a collaborative research process, involving the research team and researcher to achieve richer interpretation of meaning rather than consensus of meaning (Byrne, 2021). (p. 5)

That authors deploying methodologically *congruent* practices explain them around what they are *not* doing suggests the broader context remains one in which positivist/realist-founded practices remain normative and expected (see Varpio *et al*., 2017, 2021). Reviewers and editors have an important role to play here, by not expecting authors to explain their departure from positivist/realist norms, and we encourage editorial intervention to support authors in this, if reviewers have asked for such explanation from authors.

In some papers, the *stated* theoretical position (e.g. constructionism; relativism) was (theoretically) incongruent with reported research practices (data saturation; practices to ‘minimize bias’). Such practices are *not* incongruent with all forms of TA (though they *are* incongruent for reflexive TA), highlighting the importance of reflexive openness and explicit and knowing theoretical positioning as a crucial consideration for quality TA, regardless of approach. In our experience, some of this incongruence can be introduced *through* (unknowing or required) adherence to popular quality and reporting checklists—such as COREQ (Tong *et al*., 2007), which a few cited. If used ‘unknowingly’, such quality and reporting tools can foster incongruence through universalizing theoretically delimited constructs (e.g. member checking) and using positivist/realist and non-positivist concepts interchangeably (e.g. researcher bias and reflexivity).

### Examples of good practice in discussing theoretical assumptions

There were some good examples where the conceptual underpinnings of the research were discussed. In research exploring the gambling practices of younger women in Australia, Thomas *et al*. (2022, p. 3) described their critical qualitative approach as:

acknowledge[ing] the role of power, inequality, and injustice in health and social issues (Charmaz, 2017; Jacobson and Mustafa, 2019). […] While traditional qualitative methodologies aim to interpret the world, critical qualitative inquiry aims to change the world (Denzin, 2017). For the present study, this meant putting the voices of women at the centre of inquiry in order to reveal opportunities for social change, activism and policy reform (Denzin, 2017).

And in their study of meaning-making around warning labels on alcoholic beverage containers (also in Australia), May *et al*. (2022, p. 4) discussed their conceptualization of language:

Two key assumptions underpin our analysis. First, that the cultural and social significance of alcohol is socially produced and reproduced through language (Harré and Van Langenhove, 1991); therefore, shared social experiences are drawn upon to re-construct (not just describe) social reality (Potter, 1996b; Andrews, 2012). Second, the meaning of language is context-specific and interpretations will vary depending on the nature of the discussion (Potter and Wetherell, 1987).

These examples demonstrate the possibility of cogently yet succinctly describing the theoretical assumptions that underpin an analysis. More recently published papers in *HPI*—published after we selected the ‘sample’ for our review—provide further examples of good practice in engaging with theory in reflexive TA research. Reporting research exploring COVID-19 vaccine decision-making during pregnancy in Aotearoa/New Zealand, Jones and Neely (2023) discussed the poststructuralist approach informing their study and their use of the story completion method:

With a post-structuralist lens, story completion then becomes a method for examining the knowledge systems and discourse which inform participants’ thinking […] Story completion deliberately aims to not uncover personal views or experiences of the study’s participants, rather, the participant is analysed as a complex and variable function of discourse (Gravett, 2019). By adopting a post-structuralist approach, we were able to examine the stories through discourse, tropes, constructions or discursive repertories that inform participants’ understanding and decision-making (Gravett, 2019). (p. 4)

Story completion, like many other data generation methods, can be theorized in different ways with regard to what the data give researchers access to—in this instance, social discourses. For this reason, it is important that researchers anchor their use of a method in a clearly demarcated theoretical framework. Walsh *et al*. (2023), in a study exploring Irish young people’s perspectives on school-based mental health and suicide prevention using a participatory approach, noted their ‘critical view’ (p. 2) on COREQ and their rejection of elements not coherent with reflexive TA. They described the ‘critical paradigm’ informing their research and their assumption that ‘individuals exist within power-laden and inequitable environments, which means that power is always at play’ (p. 2)—an assumption that they argued has important implications for researching young people’s perspectives on mental health in school settings which ‘contain significant power imbalances between adults and young people’ (p. 3). They also noted their contextualist and critical approaches to epistemology:

we believe that reality is shaped by social, political, cultural, economic, ethnic and gender values. Research from a critical perspective strives to target issues in social life, such as social justice and marginalism while considering issues of power in the research process. (p. 3)

These assumptions informed their use of a participatory approach to inquiry, one that:

acknowledges power as a central research component and offers a way to intervene with unavoidable notions of power, by placing enhanced value on young people’s authentic knowledge and perspectives on the world. (p. 3)

Their ontological and epistemological assumptions also informed their choice and use of reflexive TA, and their understanding of power and its centrality informed the refinement and finalization of themes. Walsh *et al*.’s account provides a particularly compelling example of researchers ‘owning’ their theoretical perspectives and assumptions, and articulating *how* these shaped their design choices and research practices.

## INCONGRUENCE IN THEME CONCEPTUALIZATION

Incongruence in the conceptualization of themes was common across the articles. Different approaches to TA conceptualize themes differently, in ways that matter for quality.

### Topic summary and meaning-based themes

We have differentiated two common approaches as *topic summaries* (or categories) and *meaning-based themes*. Topic summary themes draw together material related to a particular data topic, domain or category (e.g. barriers to implementing policy X) and summarize data content relevant to that topic. Topic summaries often map closely to research/data collection questions/areas, making such ‘themes’ *appear* to pre-exist in the analysis. Analysis becomes a process centred on allocating data to (pre-known) themes, and determining the nature of these through exploring and/or summarizing what participants said in relation to these themes (often drawing together disparate observations related to the topic). Such themes often *evoke* a realist/positivist model of research, with the themes treated as real entities, located within data, for the researcher to (accurately) ‘extract’. Topic summary themes were prevalent in the articles—even those described as using reflexive TA, where topic summaries are conceptually incongruent (yet quite common in published research; Braun and Clarke, 2021b, 2023a, 2023b).

In reflexive TA, themes are conceptualized as meaning-rather than topic-centred, and, importantly, developed *from* and *through* data coding, and only *after* considerable analytic engagement. They have no ontological existence separate from the analytic process. This means when doing reflexive TA, it is not possible to ‘code for themes’ or to use coding to ‘allocate data to themes’ because themes cannot be pre-identified. Themes are interpretative stories about data, stemming from the researcher’s subjectivity, and crafted through their rigorous but positioned reading of the data. (We use stories here not to evoke the ‘made up things’ sense, but rather to convey the sense of there being a key message each theme is trying to convey.) Rather than capturing entities *within* the data, identified by the researcher, themes offer ‘takes’ on the data. Shared meaning themes should be rich and multi-faceted, encompassing multiple observations about the central concept (uniting meaning) of the theme. We have found a dandelion seed head offers a useful visual analogy (Braun and Clarke, 2022): it has a calyx (central concept) and numerous pappus (facets of the central concepts) all connected to the central calyx. Although it can be difficult to avoid writing about themes or meaning as if they exist *in* data, we encourage researchers reporting reflexive TA to use more subjective and productive language to evoke this creative process (Finlay, 2021)—themes are developed, produced, crafted, created, constructed rather than identified, found or discovered, or emerging from data, like Venus arising from the sea fully grown in Botticelli’s famous painting ‘The birth of Venus’.

### Examples of good practice in theme conceptualization and development in reflexive thematic analysis

Given that theme mismatching is common in TA research, we provide two examples of good reflexive TA themes from the articles reviewed. The themes from McGrath *et al*.’s (2022) research exploring the experiences of participants in the health and wellbeing initiative Sheds for Life (SFL) in Men’s Sheds settings in Ireland, told a story of three key elements of change:

- *creating the right environment* highlighted the importance of creating a supportive environment to facilitate men engaging optimally with SFL;
- *normalizing meaningful conversations, a legacy for talking health* conveyed the impact of SFL in encouraging meaningful conversations about health and wellbeing. Key aspects were conveyed with two subthemes: *creating safety and trust* (how SFL built on the safety and intimacy within the Shed context); *strengthening bonds* (how SFL deepened the sense of connection within the Sheds);
- *transforming perceptions of how men do health* explored how SFL was gender transformative, with participants reframing what it meant for them as men to be healthy. Subthemes *reaping the benefits of engaging with health*, and *reframing attitudes towards health* conveyed core elements of this transformation.

In their research, information relevant to health promotion, such as ‘barriers’ and ‘facilitators’ (as mentioned, a common focus in the reviewed papers), *was* discussed within and across the themes. However, these did not *structure* the analysis—they were not used as either themes or subthemes. The barrier of cost was briefly noted in *creating the right environment*, and that theme effectively focused *on* a ‘facilitator’—the right environment. But the theme centred the story, rather than the category. We note this to show that within reflexive TA, it is possible to address such core considerations for health promotion, without structuring the analysis around them (by presenting a summary of information related to each). This is a key way the ‘output’ of reflexive TA typically differs from something like framework analysis—highlighting that understanding your research output ‘needs’ is important for determining which version of TA suits your project. In several of the reviewed papers, an approach like framework analysis (Smith and Firth, 2011) seemed like it would have offered a better fit: an approach designed for research with predetermined information needs, reporting topic summary themes, often known in advance, and a hierarchal thematic structure used to parse out the different experiences and viewpoints expressed within the data.

Freeman *et al*. (2017) reported four meaning-based themes to capture core aspects of what participants reported gaining from outdoor/rural walking and solo experience exercises:

(1) *gaining a sense of freedom and escape;*
(2) *gaining a sense of awareness and sensitivity to one’s environment and its influence;*
(3) *gaining confidence in being able to cope and take action;*
(4) *gaining a sense of perspective on and an appreciation for life.*

We simply report the theme *names* to highlight how informative these can be in conveying the central concept of the theme—and to signal how a strong story theme (name) can entice the reader in, piquing curiosity.

The relatively small number of reported themes for both studies allowed for depth and complexity, and multifaceted themes. In contrast, some of the reviewed papers reported a large number of ‘themes’ and subthemes (e.g. 20–30 in some papers), and/or elaborate and often fragmented thematic structures, with multiple theme levels (including themes, categories, subthemes and/or codes). Such themes were often categories/topic summaries; subthemes were often single-faceted, more akin to codes in reflexive TA. Given word count constraints, there is a practical limit to the amount of depth and detail that can be provided when reporting such a large number of themes. In some coding reliability and codebook approaches, including framework analysis, as noted above, a differentiated thematic structure with subthemes nested within themes, and perhaps themes nested within overarching themes, is understood as a way to capture the complexity of the data being analysed. In reflexive TA, a highly differentiated thematic structure can work against complexity and the development of unifying concepts and meanings. Complexity in reflexive TA is evident in the presentation of rich and multifaceted themes, with subthemes used judiciously to highlight a dimension of the central concept of the theme (subthemes *aren’t* a necessary feature of a reflexive TA). The difference in thematic structure and what it means across different TA approaches again highlights the importance of understanding and knowingness for quality and congruent TA reporting.

### Qualitative data analysis software

Qualitative data analysis software (QDAS) was used in many reviewed papers, and such tech *may* have facilitated some papers reporting high numbers of themes, with highly differentiated thematic structures. No coding technology—whether QDAS or paper and pencil—is neutral—they all shape how researchers engage with and analyse data (e.g. QDAS may facilitate over-coding; hand coding *under*-coding). If doing reflexive TA specifically, where themes are meaning-based stories and subthemes convey important parts of the story, knowingness around the process, and the *purpose* of coding, is really important to avoid incongruence and an elaborate, fragmented, topic summary output.

## RECOMMENDATIONS FOR DOING AND REPORTING METHODOLOGICALLY CONGRUENT THEMATIC ANALYSIS RESEARCH

We end with eight recommendations to enhance methodological congruence. Although addressed to researchers seeking to conduct and publish high-quality TA research, we encourage reviewers assessing manuscripts submitted to *HPI* to consider these when TA has been used.

1. Read methodological literature—do not cite without reading.
2. Develop a sound (good enough) understanding of the diversity within TA. If ‘mixing’ from different approaches, ensure these are compatible, and explain why; if developing your own idiosyncratic TA approach, explain why this was necessary, and what it allowed you to do that other approaches didn’t.
3. Reflect on your research values and the goals and purpose of your research (e.g. more open and exploratory [reflexive TA] versus more delineated and predetermined [codebook or coding reliability]). Select an appropriate TA approach, and justify your use of this approach.
4. Own your perspectives. Discuss the theoretical assumptions anchoring your research (with reference to relevant literature), clarifying or explaining *your* use of terms with multiple meanings. Acknowledge and reflect on researcher subjectivity and your role in knowledge generation—in supplementary materials if necessary.
5. Describe *how* exactly you implemented your approach to TA (both the practical aspects and any choices that needed to be made, such as a semantic and/or latent approach to coding).
6. Ensure your conceptualization of themes (topic summaries or shared meaning) is congruent with your approach to TA.
7. Use quality practices that are methodologically congruent with your research values and TA approach (e.g. in reflexive TA, positivist/realist practices like participants validating the ‘accuracy’ of transcripts and data interpretation, and researchers establishing inter-coder reliability, are not congruent. Member reflections [Tracy, 2010], which offer opportunities for collaboration and reflexive elaboration, and co-researchers acting as ‘critical friends’ [Smith and McGannon, 2018] to encourage reflexivity, are congruent.).
8. Be cautious of quality and reporting checklists and standards; evaluate for methodological incongruence before simply using ‘recommended’ criteria.

In order to support researchers using reflexive TA, and reviewers evaluating papers using TA, we recently developed some Reflexive Thematic Analysis Reporting *Guidelines* (RTARG) (Braun and Clarke, 2024); we use guidelines rather than checklist or standards to emphasize a less prescriptive approach. These are intended to offer a methodologically congruent alternative to reporting checklists like COREQ, which can introduce incongruence. We have also written more widely about good and problematic practice in doing and reporting reflexive TA (e.g. Braun and Clarke, 2021b, 2023a, 2023b), and we particularly encourage reading beyond our original 2006 paper. And for those using *other* forms of TA, we encourage similarly thoughtful engagement with methodologically congruent research and reporting practices.
